# 

**Published:** 1897-12

**Authors:** 


### CONTENTS.

ORIGINAL ARTICLES: PAGE
Dairy- and Milk-Inspection. By C. COURTNEY McLEAN, V.S. . . . . . 745
Tuberculosis and Milk-Supply. By MAZYCK P. RAVENEL, M.D. . . . . . 753
The Close Relationship Existing Between the Veterinary Profession of To-day and Organized Societies for the Prevention of Cruelty to Animals. By W. H. DALRYMPLE, M.R.C.V.S. . . . . . . . . . . . . . . . 762
The Value of Exercise. By J. CURTIS MICHENER, V.S. . . . . . . . . 765
Acute Indigestion in the Cow. By W. H. RIDGE, V.M.D. . . . . . . . 769
Interesting Tuberculin Reactions. By F. S. SCHOENLEBER, M S.A., D.V.S. . . 771
Cystic Disease of the Antrum. By J. L. CLARK, V.S. . . . . . . . . 773

ABSTRACTS FROM FOREIGN JOURNALS:
Number of Veterinarians in the Different European Countries . . . . . . 774
A Case of Traumatic Emphysema . . . . . . . . . . . . 774
Hygroma of the Subcutaneous Bursæ on the Ischiatic Promontories in Cattle . . 775

SELECTIONS . . . . . . . . . . . . . . . . . . . 777

REPORTS OF CASES:
Intussusception of Small Intestine of Colt; Operation; Recovery. By E. MAYHEW MICHENER, V.M.D. . . . . . . . . . . . . . . . 779
Intestinal Perforation by Tænia Serrata. By HARRI H. DELL, D.V.S. . . . 780
Potash Iodide in Scirrhous Cord. By M. J. TREACY, Veterinarian, U. S. A. . . 782
Spasmodic Contractions with Hypertrophy of the Retractor Muscles of the Penis of a Bull. By D. S. WHITE, D.V.M. . . . . . . . . . . . 783
Coxitis in a Mule. By W. E. A. WYMAN, V.S. . . . . . . . . . 785

CONTROL WORK . . . . . . . . . . . . . . . . . 786

EDITORIALS:
1897—Journal—1898 . . . . . . . . . . . . . . . 787
Let the Shoemaker Stick to His Last . . . . . . . . . . . 788
Ontario Sets a Bad Example . . . . . . . . . . . . . 789

NECROLOGY . . . . . . . . . . . . . . . . . . 792

REVIEW—Diseases of Swine . . . . . . . . . . . . . . 795

SOCIETY PROCEEDINGS:
Keystone Veterinary Medical Association—Veterinary Medical Association of New York County—Veterinary Medical Society of the Ontario Veterinary College—Montreal Veterinary Medical Association—Massachusetts Veterinary Association—North Carolina Veterinary Medical Association— Genesee Valley Veterinary Medical Association . . . . . . . . . . . . 796

PERSONALS . . . . . . . . . . . . . . . . . . 803

PENNSYLVANIA STATE BOARD OF VETERINARY MEDICAL EXAMINERS. 804

INDEX . . . . . . . . . . . . . . . . . . . . 805

